# Role of Airwave Oscillometry in Patients with Combined Fibrosis–Emphysema Syndrome (CPFE) with Preserved FEV1/FVC Ratio

**DOI:** 10.3390/diagnostics16081159

**Published:** 2026-04-14

**Authors:** Raffaella Pagliaro, Filippo Scialò, Domenica Francesca Mariniello, Vito D’Agnano, Maria Ilaria Palma, Susan F. Campbell, Josuel Ora, Francesco Saverio Cerqua, Giulia Maria Stella, Andrea Bianco, Fabio Perrotta

**Affiliations:** 1 Department of Translational Medical Sciences, University of Campania ‘L. Vanvitelli’, 80131 Naples, Italy; domenica.mariniello@ospedalideicolli.it (D.F.M.); vito.dagnano@studenti.unicampania.it (V.D.); ilaria.palma90@gmail.com (M.I.P.); andrea.bianco@unicampania.it (A.B.); 2 U.O.C. Clinica Pneumologica L. Vanvitelli, A.O. dei Colli, Monaldi Hospital, 80131 Naples, Italy; fscerqua@gmail.com; 3 Department of Molecular Medicine and Medical Biotechnologies, University of Naples “Federico II”, 80131 Naples, Italy; filippo.scialo@unina.it; 4 CEINGE-Biotecnologie Avanzate Franco Salvatore, 80131 Naples, Italy; 5 Department of Advanced Medical and Surgical Sciences, University of Campania ‘L. Vanvitelli’, 80138 Naples, Italy; sfmcampbell@gmail.com; 6 Unit of Respiratory Medicine, Department of Experimental Medicine, University of Rome Tor Vergata, 00133 Rome, Italy; josuel.ora@ptvonline.it; 7 Department of Internal Medicine and Medical Therapeutics, University of Pavia, 27100 Pavia, Italy; g.stella@smatteo.pv.it; 8 Respiratory Diseases Unit, Cardiothoracic and Vascular Department, IRCCS San Matteo Polyclinic Hospital, 27100 Pavia, Italy

**Keywords:** CPFE, spirometry, airwave oscillometry, small-airway disease

## Abstract

**Introduction:** Emphysema is frequently observed in patients with fibrosing interstitial lung diseases (f-ILDs), leading to the clinical entity known as combined pulmonary fibrosis and emphysema (CPFE). This study aimed to evaluate the utility of airwave oscillometry (AOS) in detecting small-airway dysfunction (SAD) in patients with CPFE. Due to the coexistence of both restrictive and obstructive airway disease, spirometry is comparatively less sensitive in detecting airflow limitations in this population. **Methods:** A cohort of 52 patients with CPFE was recruited from Monaldi Hospital, Naples, between January and September 2023. Pulmonary function tests—including spirometry, body plethysmography, and single-breath diffusing capacity for carbon monoxide (DLCO)—were performed at baseline and following bronchodilator administration. Patients with normal FEV1/FVC ratios underwent airwave oscillometry (AOS) to assess respiratory system resistance (Rrs) and reactance (Xrs), with SAD defined as an R5–R19 value greater than 0.07 kPa·s·L^−1^. **Results:** AOS-defined SAD was present in 40.4% (21/52) of the cohort. The R5–R19 value in the SAD group was 0.13 ± 0.05 kPa·s·L^−1^, which can be compared to 0.04 ± 0.02 kPa·s·L^−1^ in patients without SAD. Patients with SAD were more likely to be undergoing maintenance bronchodilator therapy (16/21; 76.2%) than those without SAD (8/31; 25.8%) (*p* = 0.015). Fourteen CPFE patients met the criteria for bronchial responsiveness. CPFE patients who responded to bronchodilators had lower R5-R19 values than non-responders (0.04 ± 0.02 vs. 0.09 ± 0.06 kPa·s·L^−1^; *p* = 0.04). **Discussion:** Although AOS parameters did not significantly change following bronchodilator administration, this study underscores the value of AOS in detecting peripheral airway dysfunction, which may be under-recognized by conventional spirometry. **Conclusions:** AOS shows promise as a diagnostic adjunct for identifying SAD in CPFE patients and may complement standard pulmonary function testing in clinical practice. Further multicenter studies with larger cohorts are warranted to validate these findings and investigate the longitudinal impact of SAD on disease progression and treatment outcomes in CPFE.

## 1. Introduction

Emphysema is relatively common in patients with fibrosing interstitial lung diseases (f-ILDs), including idiopathic pulmonary fibrosis (IPF) or other idiopathic or secondary ILDs, delineating an entity known as combined pulmonary fibrosis and emphysema (CPFE) [1,2,3]. The prevalence of CPFE varies across different populations, ranging from 8 to 67% of patients with IPF [4], while its prevalence in the general population remains unknown. CPFE was originally defined based on clinical, physiological, and high-resolution computed tomography (HRCT) imaging features [2]. The emphysematous component of CPFE is assessed via HRCT, with diagnostic thresholds varying from >0% to >15% of total lung volume [5,6]. However, despite preserved airflow and lung volumes, patients show reduced exercise capacity and severely impaired diffusing capacity for carbon monoxide (DLCO) [7,8]. Compared to those with isolated IPF, patients with CPFE have greater lung volumes and a typically normal or slightly reduced FEV1/FVC ratio [9,10], which is generally lower than that in IPF alone. In contrast to Chronic Obstructive Pulmonary Disease (COPD), CPFE patients tend to have relatively preserved forced expiratory volume in one second (FEV1) values and FEV1/FVC ratios, less lung hyperinflation, and more severely reduced DLCO [11,12]. In CPFE, flows and volumes often remain stable due to opposing mechanical influences: fibrosis increases lung stiffness and helps keep airways open during exhalation, while emphysema tends to reduce airway support [13,14]. As fibrosis advances, this balance can lead to a ‘normalization’ of the FEV1/FVC ratio, even though patients may experience worsening breathlessness and declining gas exchange capacity [1,14]; however, in advanced disease, respiratory support may be required [15,16,17]. In addition, ILD can complicate the risk–benefit assessment of systemic immunomodulatory therapies [18,19]. As a result, there are no parameters whose ability to monitor disease progression in CPFE has been validated. Changes in FVC, commonly used to monitor IPF progression [20,21], are unreliable indicators in patients with CPFE [8]. Moreover, the small airways play a key role in the disease’s symptoms, such as dyspnea and exercise limitation [22]. In some cases, spirometry may still show relatively normal lung function, while small-airway impairment can already be present, potentially contributing to disease progression and worsening symptoms [23]. However, spirometry is the most used method of assessing small-airway function, using parameters such as FEF25–75%, FEF50%, and FEF75%. Nevertheless, this technique requires good patient cooperation, and the variability in results can impact the reliability of these measurements. Despite the lack of reliable spirometry markers, there is growing interest in the use of the forced oscillation technique (FOT) as a sensitive and non-invasive tool for detecting early changes in the respiratory system, especially in the small airways, before they are visible via spirometry [24]. Recent translational studies on fibrotic ILDs have highlighted biologic hallmarks linked to disease severity—including cellular-senescence-related pathways [25,26] and systemic and metabolic signaling [27,28,29,30]—supporting the need for sensitive physiologic tools beyond conventional spirometry. Moreover, the expanding and increasingly personalized therapeutic landscape in ILDs further emphasizes the value of non-invasive methods suitable for repeated functional phenotyping in complex entities such as CPFE [31,32,33]. Small airways (with internal diameters < 2 mm) contribute to a small portion of total airway resistance due to their large cross-sectional area. The aforementioned technique measures the mechanical properties of the respiratory system by applying oscillatory signals to the airways and measuring the response in terms of resistance (Rrs) and reactance (Xrs) [34,35,36]. Various commercial devices have been developed for airway oscillometry, employing different technologies such as impulse loudspeaker sources or vibrating mesh systems. Among these, vibrating mesh technology appears to offer greater sensitivity in detecting changes in reactance, especially in patients with airflow obstruction [37].

The aim of this study was to evaluate the role of airwave oscillometry (AOS) in detecting small-airway dysfunction (SAD) in patients with CPFE. We investigated the correlation between the presence of SAD and lung function parameters. Furthermore, we assessed differences in bronchodilator responsiveness between patients with and without SAD and evaluated whether AOS-derived indices differed between groups.

## 2. Materials and Methods

### 2.1. Study Design

This was a single-center observational study conducted at the Clinic for Rare Pulmonary Diseases, Monaldi Hospital, Naples, between January and September 2023. Fifty-two patients diagnosed with CPFE with preserved FEV1/FVC ratios (>5th percentile) were enrolled according to our research statement [1]. At the time of enrollment, all patients underwent a comprehensive assessment including spirometry (with dynamic volume assessment) and pletismography (for static volume) in accordance with the American Thoracic Society (ATS) and European Respiratory Society (ERS) recommendations. Additionally, single-breath DLCO was measured. AOS was performed using the tremoFlo^®^ c-100 device (Thorasys Thoracic Medical Systems Inc.6560 Esplanade Avenue, Suite 103 Montreal, QC H2V 4L5 Canada), with three consecutive measurements taken, as per to ERS guidelines. All pulmonary function tests were conducted at baseline and after 15 min of administering 400 mcg of Salbutamol, a short-acting B2-adrenergic bronchodilator. Bronchodilator response was defined as a >12% and 200 mL increase in FEV1 (flow response) or a 10.5% and 320 mL increase in FVC (volume response) [38,39]. Other parameters were also evaluated, including anthropometric data (weight, height, and BMI), use of home-oxygen therapy (continuous and/or intermittent, i.e., during exercise or at night), ongoing antifibrotic treatment (pirfenidone or nintedanib), and baseline bronchodilator therapy. Arterial blood gas analysis was performed, and disease severity was assessed using the GAP index (a multiparametric score incorporating sex, age, FVC, and DLCO) [40]. The degree of dyspnea was evaluated using the modified Medical Research Council (mMRC) questionnaire. The study was conducted in accordance with the Declaration of Helsinki, and the protocol was approved by the Local Department-of-Ethics Committee. Informed consent was obtained from all subjects involved in the study.

### 2.2. Airwave Oscillometry (AOS)

Measurements were obtained using a TremoFlo C-100 device (Airwave Oscillometry System™, Thorasys, Montreal, QC, Canada; software version 1.0.34.32), with standard multi-frequency signal settings ranging from 5 to 37 Hz, suitable for adult populations. To ensure accuracy, a calibration check of resistance was performed daily using a 0.2 kPa·s·L^−1^ reference mesh. Participants remained seated upright, wearing a nose clip and stabilizing their cheeks with their hands to minimize upper-airway shunting. They maintained a tight seal around the mouthpiece while receiving a multifrequency sinusoidal signal delivered via a loudspeaker for 16 s of relaxed tidal breathing. At least three consecutive, technically acceptable measurements were collected per subject. Any segments affected by artifacts were excluded based on visual inspection. Key parameters derived included resistance at 5 Hz (R5), resistance at 19 Hz (R19), the difference between R5 and R19 (R5–R19), reactance at 5 Hz (X5), and the area of reactance (AX). Measurement reproducibility was ensured by confirming a coefficient of variation (CV) for respiratory impedance (Zrs) below 15%, based on a minimum of three valid recordings.

### 2.3. Definition of the Criteria for Small-Airway Disease (SAD)

SAD was defined using IOS as a difference in resistance at 5 Hz and 19 Hz (R5–R19) greater than 0.07 kPa·s·L^−1^ [41]. We considered a reduction in resistance from 5 to 19 Hz (R5–R19, in kPa·s·L^−1^) indicative of the peripheral airway resistance index and an R5-R19 value greater than 0.07 kPa·s·L^−1^ indicative of the presence of SAD.

### 2.4. Statistical Analysis

Statistical analysis was performed using Jamovi software (version 2.3). Categorical or qualitative variables were expressed as counts and percentages, while continuous or quantitative variables were presented as medians and interquartile ranges (IQRs) or means and standard deviations (SDs), depending on their distribution. For univariate correlations, the Pearson correlation coefficient was used. A significance level of *p* < 0.05 was employed.

## 3. Results

In our study, we enrolled 52 patients who had been diagnosed with CPFE with a preserved FEV1/FVC ratio. Overall, forty-four were male (84.6%) and eight were female (15.4%). Baseline demographic, functional, AOS, and arterial blood gas data are summarized in Table 1. The mean pre-bronchodilator FEV_1_ was 88.73 ± 17.81% predicted, and the mean FVC was 81.08 ± 17.08% predicted, with a preserved FEV_1_/FVC ratio (84.26 ± 6.45). Gas-exchange impairment was marked (DLCO 37.45 ± 17.10% predicted), and 34 patients (65.4%) required oxygen supplementation at rest or intermittently.

The cohort was then divided into two groups based on the presence or absence of SAD, as mentioned above. According to our analysis, twenty-one patients out of fifty-two (40.4%) were identified as having CPFE with SAD. Twenty-one of 52 patients (40.4%) were classified as having SAD. Group comparisons are reported in Table 2. The patients with and without SAD did not differ significantly in age (72.40 ± 3.73 vs. 72.29 ± 4.60 years; *p* = 0.623), male sex prevalence (16/21 vs. 28/31; *p* = 0.531), GAP index (3.75 ± 1.39 vs. 4.71 ± 1.54; *p* = 0.160), or dyspnea burden (mMRC ≥ 2: 12/21 vs. 16/31; *p* = 0.856). Oxygen supplementation showed a non-significant trend toward being more frequent in the non-SAD group (7/21 vs. 25/31; *p* = 0.070). Antifibrotic treatment rates were comparable between groups (16/21 vs. 20/31; *p* = 0.856), whereas the use of maintenance bronchodilators was significantly more common in the SAD group (16/21 vs. 8/31; *p* = 0.015).

**Figure 1:**
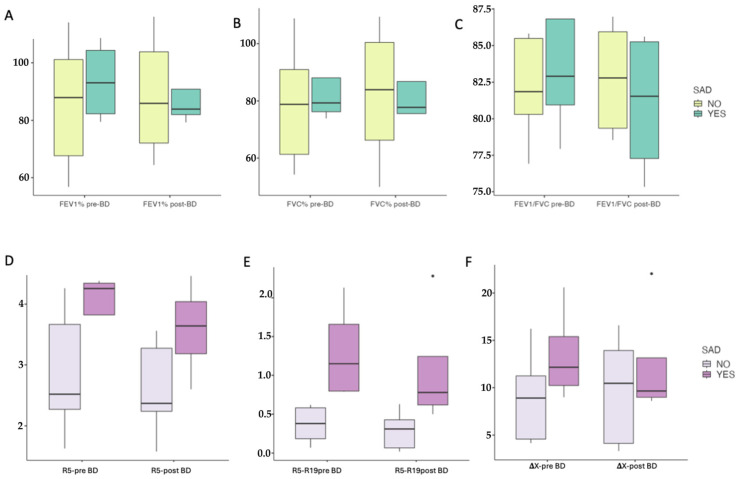
Comparison of pulmonary function tests between patients with and without airway dysfunction (SAD). (**A**–**C**) pre- and post-bronchodilatator values for FEV1% predicted, FVC% predicted, and FEV1%/FVC ratio, respectively. (**D**–**F**) pre- and post-bronchodilatator values for R5, R5–19, and AX, respectively.

Pre- and post-bronchodilator pulmonary function comparisons are reported in Table 3 and illustrated in Figure 1. Spirometric indices were similar between the SAD and non-SAD patients both before and after bronchodilator administration, with no significant between-group differences in FEV_1_ or FVC (all *p* > 0.05). In contrast, AOS parameters consistently distinguished the two phenotypes. Compared with non-SAD patients, those with SAD showed higher R5, R5–R19, and AX at baseline (R5: *p* = 0.003; R5–R19: *p* < 0.001; AX: *p* = 0.031), and these differences persisted after bronchodilation (R5: *p* = 0.031; R5–R19: *p* < 0.001; AX: *p* = 0.026). The bronchodilator-induced changes were modest and did not differ significantly between groups, with a similar variation in R5 (SAD: −0.08 ± 0.09 vs. non-SAD: 0.01 ± 0.21) and AX (SAD: −0.085 ± 0.16 vs. non-SAD: 0.028 ± 0.24).

**Figure 2:**
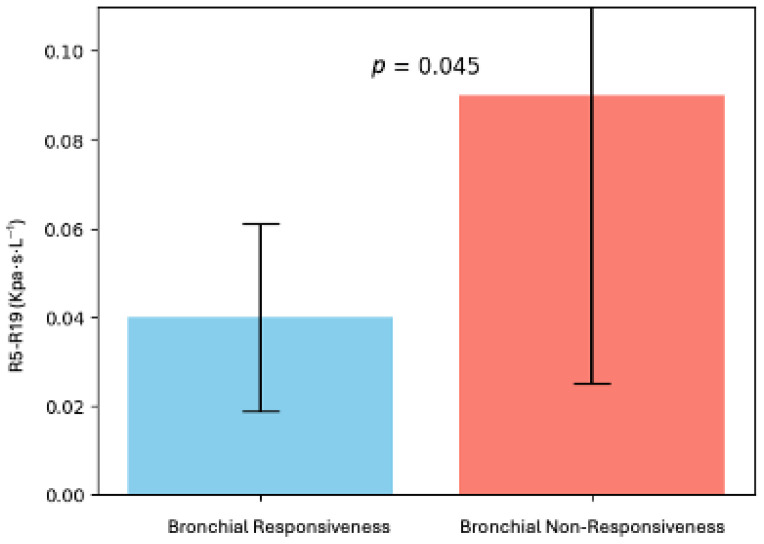
Comparison of R5-R19 values between CPFE patients with bronchial responsiveness.

Finally, in our study population, we found fourteen CPFE patients who met the criteria for bronchial responsiveness. Interestingly, the CPFE patients with bronchial responsiveness after SABA had lower R5-R19 values than the patients who did not show significant changes after the SABA (0.04 ± 0.02 Kpascal/sec/L^−1^ versus 0.09 ± 0.06 Kpascal/sec/L^−1^; *p*= 0.04) (Figure 2).

## 4. Discussion

In this study, we aimed to investigate the presence and clinical implications of SAD in patients with CPFE. Our findings indicate that SAD is prevalent in CPFE patients and associated with specific clinical features, including bronchodilator responsiveness. In our cohort of 52 CPFE patients, 40.4% (21/52) exhibited AOS-defined SAD. This aligns with previous studies on COPD, where increased peripheral airway resistance was associated with more severe airflow obstruction and lung hyperinflation [42], reflecting the recognized clinical heterogeneity of COPD [43]. Moreover, several studies have demonstrated a correlation between FOT parameters—particularly R5 and X5—and spirometry indices such as FEV1 and FVC in patients with obstructive lung diseases, including asthma and COPD [44]. Importantly, CT-based evidence pertaining to COPD suggests that oscillometric abnormalities have a clear structural counterpart, with R5–R20 tracking forms of airway remodeling such as wall thickening and airway narrowing across bronchial generations [45]. However, many authors have also pointed out that spirometry alone may lack the sensitivity required to detect small-airway dysfunction [26,46]. Many studies have suggested that spirometry is not sensitive enough to assess small-airway function [47,48]. This notion is further reinforced by observational data from symptomatic subjects with preserved pulmonary function, in whom IOS—especially R5—can help identify SAD respiratory changes even when conventional spirometry remains; notably, combining IOS with machine-learning approaches has been reported to improve diagnostic discrimination beyond any single oscillometric index [49,50,51,52,53,54]. When patients were classified into groups with and without SAD, the FOT parameters previously linked to small-airway function showed significant differences between the groups. R5, which reflects the total resistance of both central and peripheral airways, may increase due to elevated resistance in the distal airways [55]. R5–R20 serve as indicators of peripheral airway resistance and regional ventilation heterogeneity [56]; their frequency dependence is generally not observable in healthy individuals. AX is associated with peripheral lung compliance and the openness of the small airways [57]. This finding is consistent with studies on COPD patients, in whom elevated R5–R20 values correlated with worse respiratory reactance and spirometry results [44,58]. Moreover, in ILDs, FOT markers of peripheral airway dysfunction have been associated with worse respiratory quality of life [59], and reactance indices identified impaired patient-reported outcomes even when spirometry did not show clear obstructive or restrictive patterns [60,61].

However, we did not observe significant changes in R5 or reactance area (AX) before or after bronchodilation, indicating that AOS parameters may not fully capture the dynamic response to bronchodilators in CPFE patients [62]. The presence of SAD in CPFE patients underscores the need for comprehensive pulmonary function assessments beyond standard spirometry [42,63]. Notably, in our study, we found that a CPFE patient who showed a bronchodilator response after SABA had lower R5–R19 values. This might indicate that in CPFE patients, the absence of an airway response to bronchodilators can be accompanied by increased small-airway dysfunction for mechanisms that make the airway less sensitive to bronchodilators, while subjects who have features indicating bronchial responsiveness might have less small-airway involvement. While spirometry remains a cornerstone in diagnosing airflow obstruction, AOS provides additional insights into peripheral airway involvement. Our study suggests that incorporating AOS into routine evaluations could enhance the detection of SAD and lead to more-targeted therapeutic strategies. AOS evaluation, thanks to its great applicability in clinical contexts and ease of execution even for poorly compliant subjects, should be extended into clinical practice [64]. This would allow discrimination between patients with SAD (who would benefit from the use of inhaled bronchodilator therapy) and patients without SAD (for whom the use of an inhaled bronchodilator could be omitted).

### Study Limitations

Our study has several limitations. First, the sample size was small, and there was no control group. Prospective studies with a large sample size and more suitable scores for symptoms are needed to further explore the application of AOS in diagnosing and monitoring SAD in patients with CPFE. Also, FOT techniques may differ, and AOS and IOS are not interchangeable for this group of patients. Further validation is required. Moreover, the current criteria used to define SAD and bronchodilator reversibility have not been specifically studied or validated in CPFE patients. This is a critical limitation, as standard thresholds may not accurately reflect pathophysiological changes in this patient population. Notably, there is a lack of published data on R5–R20 variation after bronchodilator testing in patients with CPFE, leaving a significant gap in the literature. Future investigations should focus on establishing standardized AOS criteria for SAD in CPFE disease and clarifying how oscillometric parameters respond to bronchodilator therapy. Understanding these dynamics may help guide more personalized treatment strategies and improve disease monitoring in CPFE patients.

## 5. Conclusions

This study shows that SAD is common in patients with CPFE and can be effectively detected using AOS, even when spirometry characteristics appear normal. Patients with SAD had greater peripheral airway resistance and poorer responses to bronchodilators, and they used inhaled therapies more frequently. AOS was shown to be a useful, non-invasive tool for identifying functional impairments not captured by standard tests. These findings may support the integration of AOS into routine assessments of CPFE, although larger studies are needed to validate its clinical utility and establish specific diagnostic thresholds.

## Figures and Tables

**Table 1:** Baseline characteristics of patients included in this study. The data are presented as means ± SDs or absolute numbers and percentages.

CFPE Patients		n.52	
Gender		Pulmonary Function Test Parameters	
Male	44 (84.6%)	FEV1% Pre-bd	88.73 ± 17.81
Female	8 (15.4%)	FEV1 (l) Pre-bd	2.18 ± 0.45
Age (Years)		FVC% Pre-bd	81.08 ± 17.08
70.8 (71.40 ± 3.93)		FVC (l) Pre-bd	2.59 ± 0.51
Smoking History		FEV1/FVC Pre-bd	84.26 ± 6.45
Former	52 (100%)	FEF 75% pre-bd	74.93 ± 14.36
mMMRC		FEF 50% pre-bd	67.66 ± 20.91
0–1	24 (46.6%)	FEF 25% pre-bd	79.89 ± 20.42
≥2	28 (53.4%)	DLCO% pre-bd	37.45 ± 17.10
GAP Stages		TLC% pre-bd	66.92 ± 12.91
Stage I (0–3)	18 (27.3%)	TLC (l) pre-bd	3.88 ± 0.96
Stage II (4–5)	26 (54.5%)	Airwave Oscillometry Parameters	
Stage III (6–8)	8 (18.2%)	R5 pre-bd (kPa·s·L^−1^)	3.21 ± 1.01
COPD treatment		R5–19 pre-bd (kPa·s·L^−1^)	0.07 ± 0.06
None	28 (53.8%)	∆R5–19 (kPa·s·L^−1^)	0.20 ± 1.85
LAMA	14 (26.9%)	Ax pre-bd (kPa·L^−1^)	10.40 ± 4.93
LABA-LAMA	10 (19.2%)	∆Ax (kPa·L^−1^)	−0.01 ± 0.22
Antifibrotics		Arterial Blood Gas Analysis Parameters	
None	16 (30.8%)	Ph	7.44 ± 0.02
Pirfenidone	17 (32.7%)	pO_2_ (mmHg)	77.94 ± 9.40
Nintedanib	19 (36.5%)	pCO_2_ (mmHg)	38.17 ± 3.27
Oxygen Supplementation		HCO^3−^ (mmol/L)	26.00 ± 2.28
Rest/Intermittent Oxygen	34 (65.4%)	Lac (mmol/L)	1.06 ± 0.70

**Table 2:** Differences in CPFE patients between the SAD and non-SAD groups.

	SAD-Group n = 21 pts	Non-SAD Group n = 31 pts	*p*-Value
Age (yrs)	72.40 ± 3.73	72.29 ± 4.60	0.623
Gender (Male)	16	28	0.531
GAP Index	3.75 ± 1.39	4.71 ± 1.54	0.160
mMRC (≥2)	12	16	0.856
Oxygen Supplementation	7	25	0.070
Use of bronchodilators	16	8	0.015
Use of antifibrotics	16	20	0.856

**Table 3:** Comparison of pulmonary function between SAD and non-SAD groups before (pre-BD) and after bronchodilator administration (post-BD). Data are presented as means ± SDs.

	Pre-BD			Post-BD		
	SAD	Non-SAD	*p*-Value	SAD	Non-SAD	*p*-Value
FEV1 L	2.32 ± 0.37	2.17 ± 0.29	0.113	2.30 ± 0.27	2.20 ± 0.32	0.210
FEV1%	90.43 ± 9.69	87.26 ± 13.87	0.737	85.47 ± 8.72	86.58 ± 12.66	0.711
FVC L	2.87 ± 0.30	2.72 ± 0.42	0.126	2.90 ± 0.31	2.81 ± 0.42	0.411
FVC%	80.55 ± 9.75	79.33 ± 12.85	0.641	85.66 ± 9.16	80.94 ± 14.58	0.157
FEV1/FVC	82.70 ± 4.75	81.90 ± 6.40	0.753	80.65 ± 6.40	82.75 ± 5.80	0.690
R5	3.21 ± 0.88	2.81 ± 0.65	0.012	3.03 ± 0.81	2.73 ± 0.87	<0.001
R5-R19	0.97 ± 0.57	0.51 ± 0.24	<0.001	0.97 ± 0.57	0.51 ± 0.29	0.002
AX	11.78 ± 4.22	9.33 ± 4.16	0.024	11.53 ± 4.65	9.85 ± 3.32	0.026

## Data Availability

The original contributions presented in this study are included in the article. Further inquiries can be directed to the corresponding authors.
